# Systems approach to characterize the metabolism of liver cancer stem cells expressing CD133

**DOI:** 10.1038/srep45557

**Published:** 2017-04-03

**Authors:** Wonhee Hur, Jae Yong Ryu, Hyun Uk Kim, Sung Woo Hong, Eun Byul Lee, Sang Yup Lee, Seung Kew Yoon

**Affiliations:** 1 The Catholic University Liver Research Center & WHO Collaborating Center of Viral Hepatitis, College of Medicine, The Catholic University of Korea, Seoul 06591, Republic of Korea; 2 Metabolic and Biomolecular Engineering National Research Laboratory, Department of Chemical and Biomolecular Engineering (BK21 Plus program), Center for Systems and Synthetic Biotechnology, Institute for the BioCentury, Korea Advanced Institute of Science and Technology (KAIST), Daejeon 34141, Republic of Korea; 3 BioInformatics Research Center, KAIST, Daejeon 34141, Republic of Korea; 4 BioProcess Engineering Research Center, KAIST, Daejeon 34141, Republic of Korea; 5 Department of Internal Medicine, Seoul St. Mary’s Hospital, College of Medicine, The Catholic University of Korea, Seoul 06591, Republic of Korea

## Abstract

Liver cancer stem cells (LCSCs) have attracted attention because they cause therapeutic resistance in hepatocellular carcinoma (HCC). Understanding the metabolism of LCSCs can be a key to developing therapeutic strategy, but metabolic characteristics have not yet been studied. Here, we systematically analyzed and compared the global metabolic phenotype between LCSCs and non-LCSCs using transcriptome and metabolome data. We also reconstructed genome-scale metabolic models (GEMs) for LCSC and non-LCSC to comparatively examine differences in their metabolism at genome-scale. We demonstrated that LCSCs exhibited an increased proliferation rate through enhancing glycolysis compared with non-LCSCs. We also confirmed that MYC, a central point of regulation in cancer metabolism, was significantly up-regulated in LCSCs compared with non-LCSCs. Moreover, LCSCs tend to have less active fatty acid oxidation. In this study, the metabolic characteristics of LCSCs were identified using integrative systems analysis, and these characteristics could be potential cures for the resistance of liver cancer cells to anticancer treatments.

Hepatocellular carcinoma (HCC) is one of the most frequently diagnosed malignancies worldwide with a particularly poor prognosis, given its resistance to currently available treatments^1^,^2^. HCC is highly prevalent in Africa and Southeast Asia, and its incidence is steadily increasing in Western countries^3^,^4^. The majority of HCC develops as a result of chronic liver injury caused by infections with hepatitis B virus (HBV) and hepatitis C virus (HCV), alcohol abuse, non-alcoholic steatohepatitis, and exposure to liver toxins such as aflatoxin and oral contraceptives. Currently available options for the treatment of advanced HCC, including chemotherapy, radiation therapy, local ablation, and anti-angiogenesis therapies, have only demonstrated limited efficacy^5^,^6^. Our poor understanding of the molecular mechanisms that control initiation, progression, and treatment refractoriness of the tumor has made HCC treatment even more difficult.

In this context, recent research efforts have focused on characterizing phenotypes of cancer stem cells (CSCs) in an effort to develop new therapeutic strategies and improve outcomes in the treatment of liver cancer. CSCs or tumor initiating cells represent a small subpopulation of cancer cells in various types of cancers. CSCs possess capabilities of self-renewal and differentiation, and are believed to cause chemo- or radio-resistance, leading HCC patients to suffer frequent tumor recurrence or metastasis^7^,^8^,^9^. Among various markers of CSCs, CD133 has been widely used as a marker for the identification of CSCs in hepatocarcinogenesis^10^,^11^. CD133, a five-transmembrane-spanning cell-surface glycoprotein, is associated with resistance to existing radio/chemotherapies^12^,^13^,^14^. In our previous studies, Huh7 cells (HCC cell line) expressing CD133 exhibited increased proliferation rates *in vivo* tumor formation, and metastatic potential compared with Huh7 cells not expressing CD133 after exposure to radiation treatment *in vitro* and *in vivo*^13^,^15^,^16^. However, major factors enhancing CSC survival following radio/chemotherapies in HCC remain unclear. We focus on CSC metabolism to address this problem because metabolism generates the energy necessary for cell proliferation. For clarity, Huh7 cells either expressing CD133 or not expressing CD133 are referred to as liver cancer stem cells (LCSCs) and non-LCSCs, respectively.

Thus, we investigated the global metabolic phenotype of LCSCs using systems biology tools, including transcriptome, metabolome and their genome-scale metabolic models (GEMs), to explore genome-wide metabolic characteristics of LCSCs compared with non-LCSCs. GEMs are computational models that mathematically describe biochemical reactions in an organism of interest, and have been widely used to simulate cell/tissue-specific metabolism^17^. As a result of the integrative analysis and experimental validation, we propose reprogrammed metabolic characteristics of LCSCs in comparison with non-LCSCs.

## Results and Discussion

### Differential growth characteristics between LCSCs and non-LCSCs

**Figure 1:**
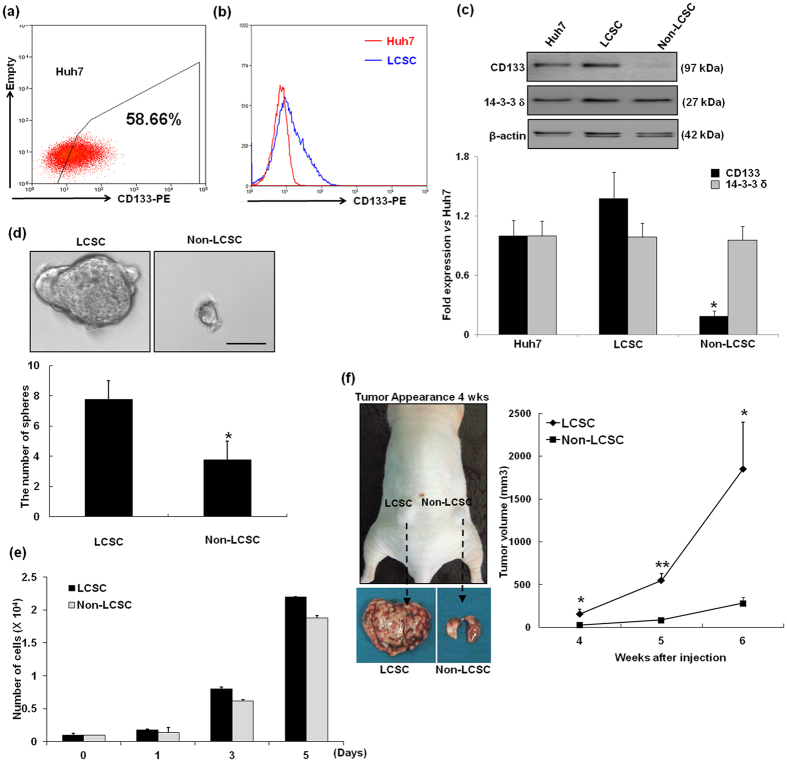
Cell sorting and characterization of tumorigenicity of LCSCs and non-LCSCs. (**a** and **b**) Optimization of flow cytometric detection of CD133 expression in hepatoma cells. The hepatoma cell line Huh7 was stained as a control for CD133 cell surface expression analysis by flow cytometry using a phycoerythrin (PE)-conjugated anti-CD133 antibody. (**c**) CD133 and 14-3-3δ expression was analyzed by Western blot using CD133 and 14-3-3δ antibody in Huh7, LCSC and Non-LCSC. Bands densities were quantified using Image J analysis software and normalized to β-actin expression. *P < 0.05 compared with Huh7 cells. (**d**) The clonogenic potential of the four subpopulations was determined using sphere formation assays. Representative image of spheres that were obtained using an inverted microscope (Olympus). Scale bar, 50 μm. The number of spheres (diameter > 50 μm) per well (2 × 10^2^ cells/well) was determined using an inverted microscope (Olympus). The results are expressed as the means ± SD of three independent experiments. *P < 0.05, LCSC *vs*. Non-LCSC. (**e**) Cell number measured by direct count of viable cells in a hemocytometer. The data for cell count/well are expressed as the mean ± SD (*n* = 5 wells at each time point). (**f**) *In vivo* tumorigenicity of the LCSCs and non-LCSCs was analyzed in Balb/c nude male mice. LCSCs and non-LCSCs were subcutaneously injected into the left and right legs of nude mice, respectively. The black arrowheads indicate subcutaneous tumors derived from either LCSCs or non-LCSCs subpopulation. Tumor volume was measured at different time points up to 6 weeks, and the average volume at each time point was plotted (n = 3; *P < 0.05 and **P < 0.001 for LCSC *vs* non-LCSC).

We utilized FACS sorting to isolate LCSCs and non-LCSCs from Huh7 cells. As a result, LCSCs accounted for 58.66% of the total Huh7 cell population (Fig. 1a and b). Considerable enrichment of CD133 expressing LCSC was observed in the positive fraction (>91% purity), as determined by FACS. Then the CD133 expression level in the isolated populations was validated by western blotting. As shown in Fig. 1c, high level of CD133 expression was detected in the LCSC, compared with non-LCSC. We previously demonstrated that the expression of 14–3–3δ, a multifunctional phosphor-serine/phosphor-threonine binding protein, was up-regulated by approximately 1.5-fold in CD133-expressing LCSCs after irradiation compared with non-exposed CD133-expressing LCSCs^15^. However, no signification difference in 14-3-3δ expression was noted between CD133-expressing LCSCs and non-LCSCs. For this reason, we chose to examine expression of 14-3-3δ protein as negative control for CSC marker. As shown in Fig. 1c, 14-3-3δ expression was similar between CD133-expressing LCSCs and non-LCSCs.

To explore the biological properties of LCSCs, we compared clonogenic potentials of LCSCs and non-LCSCs. As shown in Fig. 1d, the number of sphere-forming cells increased by at least greater than two-fold in LCSCs compared with non-LCSCs. Next, to investigate their ability to initiate tumor formation *in vitro* and *in vivo*, we performed a proliferation assay and subcutaneously injected LCSCs and non-LCSCs in both hind legs of 3 nude mice. As demonstrated by Fig. 1e, the cell proliferation was increased at various time points (1, 3 and 5 days) in LCSC compared with non-LCSC. Furthermore, after 6 weeks of engraftment, we found that LCSC injected mice had formed tumors that had a volume greater than 1300–2400 mm^3^, while non-LCSC had formed a small tumor that had a volume less than 200–320 mm^3^ (Fig. 1f). This result showed that the growth rate formed tumor in LCSC was faster than non-LCSC formed tumor and was consistent with our previously reported findings^16^. Both results showed that LCSCs exhibited a significantly increased proliferation rate compared with non-LCSCs both *in vivo* and *in vitro*.

Thus, LCSCs appeared to exhibit more stem cell-like properties, including colony formation, self-renewal and differentiation ability, and a greater ability to initiate tumors compared with non-LCSCs. Based on the observed phenotypic differences between LCSCs and non-LCSCs, we next investigated differences in their metabolism comparing their transcriptome and metabolome data to form a more detailed understanding.

### Transcriptome and metabolome data suggest that the metabolism of LCSCs is reprogrammed to increased proliferation compared with non-LCSCs

**Figure 2:**
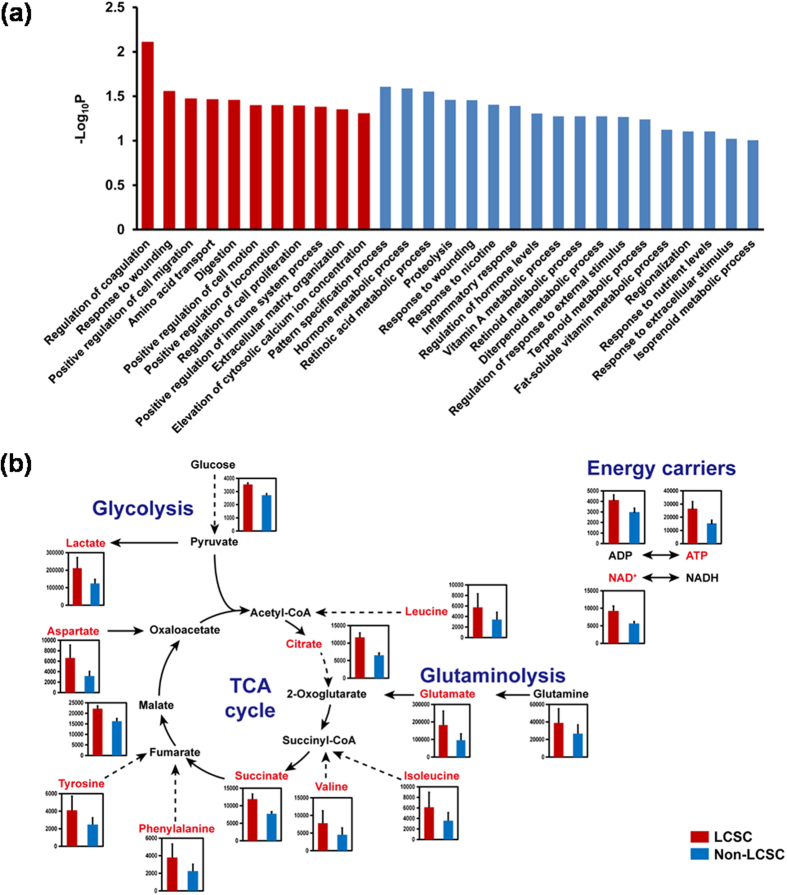
Analysis of transcriptome and metabolome data from LCSCs and non-LCSCs. (**a**) Gene ontology (GO) biological process enrichment analysis for differentially expressed genes in LCSCs compared with non-LCSCs. GO biological processes are sorted by −log_10_P. GO biological processes corresponding to up- and down-regulated genes in LCSCs are presented with red and blue bars, respectively. (**b**) Concentrations of standard metabolites (pmol/10^6^ cells) involved in central metabolism. Concentrations of metabolites involved in glycolysis, glutaminolysis, TCA cycle and energy carriers (i.e., ADP and ATP) were increased in LCSCs (red bars) compared with non-LCSCs (blue bars). Metabolites in red exhibit concentrations increased by greater than 1.5-fold in LCSCs compared with non-LCSCs. Dotted lines indicate multiple reactions. All the data samples were performed in duplicates. Error bars indicate the mean ± S.D.

Transcriptome landscapes of LCSCs and non-LCSCs were statistically compared using our transcriptome dataset previously released^16^; this comparison examining the effects of CD133 expression on the metabolism of the two types of cells without radiotherapy perturbation was not conducted in our previous study. Among a total of 18,494 genes for the cells of both cell types, the expression levels of 63 and 47 genes were significantly increased and decreased, respectively, in LCSCs compared with non-LCSCs (P < 0.05 from Student’s t-test; absolute changes >1.5-fold). These up-regulated genes in LCSCs were significantly involved in cell migration and cell proliferation according to Gene Ontology (GO) biological process categories (Fig. 2a, Supplementary Table S1 and Materials and methods). Prior studies have demonstrated that in hepatocarcinogenesis, the Endothelin 1 (*EDN1*) gene is involved in 6 out of 11 up-regulated pathways (Supplementary Table S1) and plays important roles in cell proliferation and migration by activating ERK1/2 and AKT signaling pathways^18^,^19^. This observation is consistent with biological characteristics of LCSCs, including a more rapid proliferation rate and increased metastasis compared with non-LCSCs^16^,^20^.

Metabolome analysis was subsequently conducted to directly observe differences between global metabolite concentrations of LCSCs and non-LCSCs (Materials and methods). In this metabolomic analysis, standard metabolites are the absolutely quantified metabolites (Supplementary Table S2), whereas putative metabolites represent metabolites with relative abundances in LCSCs compared with non-LCSCs (Supplementary Table S3). In this study, 48 metabolites out of a total of 110 measurable standard metabolites were detected in both LCSCs and non-LCSCs (Supplementary Table S2). For the remaining 62 standard metabolites, 51 metabolites were not detected in both cell types, and 5 metabolites were detected only in one of the two cell types. The detection of the remaining 6 metabolites was not reproducible (Supplementary Table S2). Among the standard metabolites detected in both cell types, the concentrations of lactate, citrate, succinate and 7 amino acids (i.e., aspartate, glutamate, isoleucine, leucine, phenylalanine, tyrosine and valine) were greater in LCSCs compared with non-LCSCs by more than 1.5-fold. The overall greater concentrations of lactate in LCSCs compared with non-LCSCs indicate possibly more active glycolysis in the former cell type obviously because lactate should come from glycolysis. Meanwhile, greater concentration of ATP, TCA cycle intermediates and amino acids in LCSCs suggest likely more active bioenergetics and cell proliferation capacity of LCSCs in comparison with non-LCSCs (Fig. 2b)^21^.

Similarly, additional evidence from metabolome data on putative metabolites revealed more enhanced bioenergetics of LCSCs compared with non-LCSCs. The primary evidence was derived from relatively more increased levels of phosphocreatine and carnitine in LCSCs compared with non-LCSCs. Phosphocreatine is a phosphate donor for the production of ATP, and carnitine facilitates the transport of acyl-CoA into mitochondria for fatty acid oxidation, which is also an important metabolic process for energy generation (Fig. S1 and Supplementary Table S3). In addition, 1-methylnicotinamide and *N*-acetylneuraminate were exclusively detected in the LCSCs among putative metabolites (Supplementary Table S3). Interestingly, 1-methylnicotinamide, a product of nicotinamide catabolized by nicotinamide *N*-methyltransferase (NNMT), stimulates cell growth of both rat hepatocellular carcinoma cells^22^ and human murine erythroleukemia cells^23^. *N*-acetylneuraminate, the most common sialic acid derivative in mammals, is an anticancer target because sialylation in cancer cells is associated with inhibition of apoptosis, progression, metastasis and resistance to therapy^24^. For example, a sialyltransferase inhibitor, P-3F_ax_-Neu5Ac, has a potential as an anticancer because it inhibits sialylation, leading to impaired cell adhesion, migration and tumor growth^25^,^26^.

Taken together, overall tendency of the transcriptome and metabolome profile appeared to be consistent. Integrative transcriptome and metabolome analyses revealed that metabolism of LCSCs was reprogrammed to be more proliferative based on the altered gene expressions (e.g., *EDN1*) and metabolite concentrations (e.g., 1-methylnicotinamide, amino acids, ATP, lactate, *N*-acetylneuraminate, and TCA cycle intermediates) related to cancer progression.

### MYC is a key regulator for LCSC metabolic reprogramming

**Figure 3:**
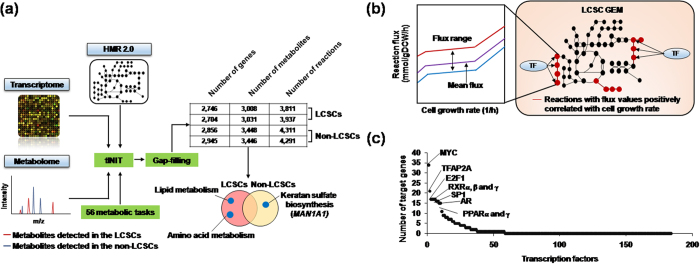
Reconstruction of LCSC and non-LCSC GEMs and application for the identification of regulators associated with cell proliferation. (**a**) Reconstruction process of LCSC and non-LCSC GEMs. Transcriptome and metabolome data were integrated with a generic human GEM, HMR 2.0^27^. During the GEM reconstruction process, draft GEMs were examined and validated by simulating the predefined 56 metabolic tasks under the cultivation condition of RPMI-1640 medium (Supplementary Table S5). The GrowMatch gap-filling algorithm was performed to enable the cell type-specific GEMs to demonstrate growth^50^. Reactions involved in lipid and amino acid metabolisms were more included in LCSC GEMs, whereas keratan sulfate biosynthetic pathway (largely mediated by *MAN1A1*) was more represented in non-LCSC GEMs. (**b**) Scheme of metabolic simulation using flux response analysis of the LCSC and non-LCSC GEMs to predict transcription factors regulating biomass generation (i.e., cell growth rate). The mean flux value (purple line) of each reaction was used when calculating the correlation between each reaction’s flux value and cell growth rate. In the right panel, upon prediction of reactions with flux values positively correlated with cell growth rate (red lines). Transcription factors (TF) that are known to bind to and regulate genes of the predicted reactions were next searched from RegNetwork^31^. (**c**) Transcription factors that potentially regulate genes responsible for the metabolic reactions with flux values positively correlated with cell growth rate. Names of the top 10 transcription factors (out of 184 transcription factors) are presented.

In addition to the analysis of transcriptome and metabolome data, we next reconstructed GEMs of LCSC and non-LCSC to further capture genome-wide metabolic differences between LCSCs and non-LCSCs. GEM is a computational model that takes into account all the known biochemical reactions based on the genomic information of the target cell type and has served as an important systems biology tool to explore genome-wide human metabolism through integration with omics data^17^. To this end, we used one of the most comprehensive generic human GEMs, HMR 2.0, along with both transcriptome and metabolome data to generate GEMs specific to LCSCs and non-LCSCs. A “task‐driven integrative network inference for tissues” (tINIT) algorithm was used for the omics data integration^27^,^28^ (Materials and methods, Supplementary Tables S4-S5 and Fig. 3a). Because the transcriptome dataset consists of duplicate samples for each LCSC and non-LCSC, a total of four functional LCSC and non-LCSC GEMs were reconstructed (Fig. 3a). See Materials and methods for details on GEM reconstruction process.

Overall, the two non-LCSC GEMs appeared to have a slightly greater model size compared with the other two LCSC GEMs in terms of the number of genes, metabolites and reactions (Fig. 3a). Close examination of the LCSC and non-LCSC GEMs revealed differences in their metabolic contents. The increased model size of non-LCSC GEMs resulted from a large number of metabolic reactions involved in glycan metabolism mostly found in the two non-LCSC GEMs, including keratan sulfate biosynthesis, *N*-glycan metabolism and chondroitin/heparan sulfate biosynthesis (Fig. 3a). Among the genes involved in glycan metabolism, the mannosidase alpha class 1A member 1 (*MAN1A1*) gene was observed, which regulates multiple metabolic reactions in the pathway of keratin sulfate biosynthesis (14 out of 43 reactions). *MAN1A1* expression level is decreased in metastatic HCC cell lines compared with non-metastatic cell lines^29^. Consistently, *MAN1A1* expression levels were decreased by 31% in LCSCs compared with non-LCSCs according to the transcriptome data. By contrast, amino acid metabolism (i.e., arginine and proline metabolism, and biosynthesis of phenylalanine, tyrosine and tryptophan) and lipid metabolism (i.e., fatty acid oxidation, and formation and hydrolysis of cholesterol esters) were largely included in the two LCSC GEMs (Fig. 3a and Supplementary Table S6). In particular, tyrosine and phenylalanine concentrations were increased by more than 1.5-fold in LCSCs compared with non-LCSCs (Fig. 2b). Such differences in metabolite contents of the LCSC and non-LCSC GEMs appeared to be consistent with the key metabolite profiles of the two types of cells (Fig. 2b), and indicate that LCSCs indeed exhibit metabolism better optimized for cell proliferation and energy compared with non-LCSC. Finally, in accordance with the metabolome data discussed above, 1-methylnicotinamide and *N*-acetylneuraminate were exclusively identified in the two LCSC GEMs.

Based on the structural differences of GEMs representing LCSCs and non-LCSCs, we next simulated the LCSC GEMs to identify metabolic reactions in LCSCs that may be highly correlated with biomass generation (i.e., increased cell proliferation rate) (Fig. 3b). Briefly, for the metabolic simulation, a simulation method called flux response analysis was conducted to identify metabolic reactions that exhibited increased flux values as the cell growth rate was forced to increase^30^ (Fig. 3b; see Materials and methods for details on metabolic simulation using flux response analysis). Subsequently, we attempted to identify major transcription factors, which may bind to and regulate genes responsible for the metabolic reactions predicted to be highly associated with biomass generation. As a result, we obtained 247 such reactions from LCSC GEMs (i.e., Pearson correlation coefficient >0.7).

**Figure 4:**
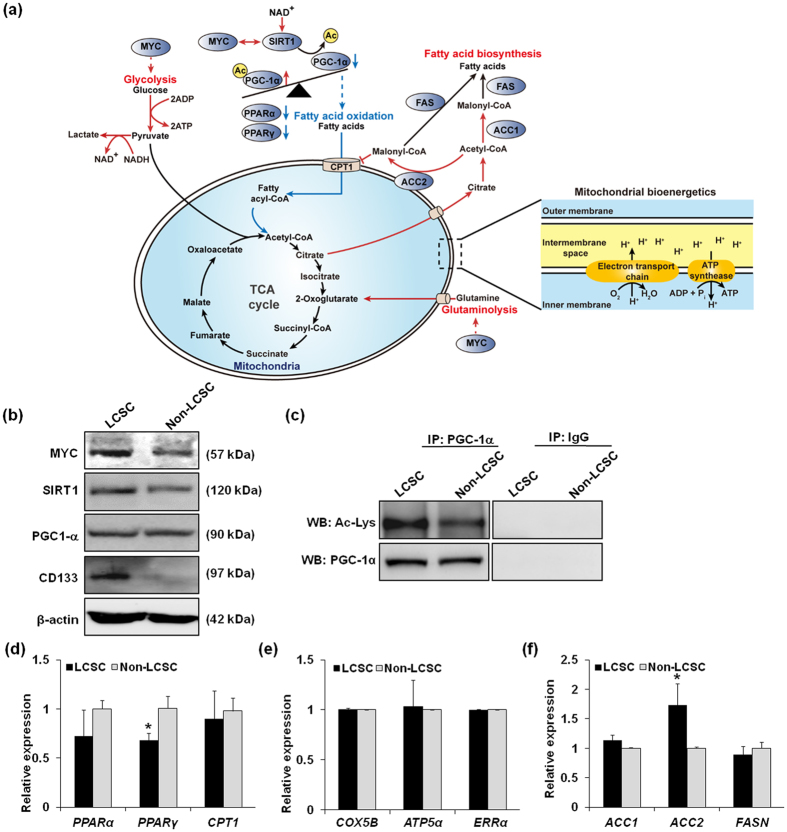
Experimental validation of reprogrammed metabolism in LCSCs in comparison with non-LCSCs. (**a**) Reprogrammed metabolism of LCSCs compared with non-LCSCs, which can be highlighted by more active glycolysis and glutaminolysis (red lines) and relatively inhibited fatty acid oxidation (blue lines). Dotted line indicates indirect regulations through signaling cascade. It should be noted that the level of acetylated PGC-1α was significantly increased in LCSCs despite high levels of SIRT1, which deacetylates PGC-1α. (**b**) Western blot analyses of MYC, SIRT and PGC-1α. Protein expression levels of MYC and SIRT1 were increased in LCSCs compared with non-LCSCs, whereas PGC-1α protein expression was comparable in both LCSCs and non-LCSCs. (**c**) Quantification of acetylated PGC-1α. Total PGC-1α was immunoprecipitated and extent of acetylation (Ac-Lys) in PGC-1α was quantified using Western blot analysis. Acetylated PGC-1α was increased in LCSCs compared with non-LCSCs. (**d**) qRT-PCR data showing mRNA expression levels of *PPAR*α, *PPAR*γ, and *CPT1* genes in LCSCs and non-LCSCs. Expression level of *PPAR*γ was significantly decreased in LCSCs. (**e**) qRT-PCR data showing comparable mRNA expression levels of *COX5B, ATP5α* and *ERRα* genes in LCSCs and non-LCSCs, which are markers for mitochondrial ATP production. (**f**) qRT-PCR data showing mRNA expression levels of *ACC1, ACC2*, and *FASN* genes responsible for fatty acid biosynthesis. Expression level of *ACC2* was significantly increased in LCSCs. Error bars indicate mean ± S.D. *P < 0.05, LCSC *vs*. Non-LCSC.

Transcription factors were next investigated by searching those that are known to bind to and regulate metabolic genes responsible for the 247 reactions. Information on transcription factors and their target genes was obtained from RegNetwork^31^ (Materials and methods). Among a total of 184 transcription factors, MYC had the greatest number of target genes that operate reactions predicted to be highly associated with biomass generation (Fig. 3c); the outcome suggests a high chance that MYC is involved in the high proliferation rate of LCSCs. Furthermore, our observations are consistent with other studies that have reported that MYC was associated with regulation of glycolytic metabolism in cancer cells and virtually all glycolytic genes^32^,^33^ (Fig. 4a). Several studies have revealed that MYC is involved in mitochondrial biogenesis and function as well as glutamine metabolism^34^,^35^. Hence, we performed experimental validation of target genes obtained by RegNetwork. As shown in Fig. 4b, the expression level of MYC was significantly increased in LCSCs compared with non-LCSCs. This result demonstrates that MYC expression in LCSCs regulated glucose metabolism and mitochondrial bioenergetics and is an important regulator of energy metabolism in the response of liver cancer to pathogenic stress. In addition to MYC, PPAR and RXR families were also predicted to regulate genes operating the biomass-associated reactions. The PPAR-RXR complex induces cell proliferation in endothelial cell proliferation and angiogenesis, and controls energy metabolism via fatty acid transport, fatty acid oxidation and adipogenesis^36^,^37^. Taken together, these results demonstrated that our systems approach is useful for understanding global metabolic characteristics of LCSCs.

### LCSCs exhibit less active fatty acid oxidation compared with non-LCSCs

We next focused on fatty acid metabolism of LCSCs and non-LCSCs because the metabolome data suggested potentially greater increased fatty acid metabolism in LCSCs compared with non-LCSCs, and the signaling factors predicted that using GEMs were highly associated with fatty acid metabolism. Previous studies demonstrated that fatty acid metabolism is often reprogrammed in cancer cells and plays important roles in biosynthesis of membrane and signaling molecules^38^,^39^. Thus, we experimentally investigated expression levels of genes and proteins involved in the fatty acid metabolism of LCSCs and non-LCSCs, thereby generating greater insight into the increased resistance of LCSCs expressing CD133 against radio/chemotherapies.

Firstly, we hypothesized that fatty acid oxidation was more active in LCSCs compared with non-LCSCs to generate more ATP for cell proliferation via oxidative phosphorylation based on the biochemical evidence of the aforementioned metabolites and signaling factors. According to metabolome data, intracellular NAD^+^ concentration was increased in LCSCs by more than 1.5-fold compared with non-LCSCs (Fig. 2b and Supplementary Table S3). Fatty acid oxidation requires NAD^+^, which induces fatty acid oxidation to generate ATP^40^. Moreover, NAD^+^ can activate the upstream signaling pathway of fatty acid oxidation by activation of SIRT1, a NAD^+^-dependent protein deacetylase (Fig. 4a). SIRT1 induces fatty acid oxidation by deacetylating its target genes in cancer^41^,^42^. In particular, PPARγ co-activator-1α (PGC-1α) is the potent target of SIRT1 and induces the transcription of target genes responsible for fatty acid oxidation by forming a complex with PPARγ and RXRα^43^,^44^. PPARγ and RXRα were also predicted in our metabolic simulation (Fig. 3c). Using Western blot analysis, we experimentally confirmed that SIRT1 protein expression was slightly increased in LCSCs compared with non-LCSCs, but whereas PGC-1α expression was comparable in both cell types (Fig. 4b and c). Given that SIRT1 is a deacetylase, we also measured the degree of acetylation of PGC-1α using immunoprecipitation experiments. In contrast to our expectation, PGC-1α acetylation levels were significantly increased in LCSCs compared with non-LCSCs, despite increased SIRT1 expression in LCSCs (Fig. 4c). This unexpected outcome potentially resulted from increased acetylation activity of other acetylases than deacetylation activity of SIRT1. We additionally measured mRNA levels of *PPARα, PPARγ*, and *CPT1* genes using quantitative RT-PCR to more thoroughly validate fatty acid oxidation. Expression levels of *PPARα* and *PPARγ* were decreased in LCSCs, supporting our conclusion that LCSCs are likely to have less active fatty acid oxidation (Fig. 4d). However, expression level of *CPT1* was comparable between LCSCs and non-LCSCs. Indeed, we want to know that increased ATP concentrations in LCSCs are produced by oxidative phosphorylation with reducing equivalents generated from fatty acid oxidation. We assessed oxidative phosphorylation activity by measuring expression levels of three genes responsible for oxidative phosphorylation; *COX5B, ATP5α* and *ERRα* genes. As a result, all of these genes were comparable in both LCSCs and non-LCSCs, indicating that ATP production through induced fatty acid oxidation did not seem to be significant in LCSCs compared with non-LCSCs (Fig. 4e). In other words, LCSCs produce more ATP using glycolysis compared with non-LCSCs (Fig. 4a).

We next focused on fatty acid biosynthesis. We expected that LCSCs exhibit increased fatty acid biosynthesis compared with non-LCSCs because rapid proliferating cells exhibit greater fatty acid biosynthesis to meet high demands for membrane biogenesis. This assumption is also supported by increased concentration of citrate in LCSCs from our metabolome data (Figs 2b and 4a). To validate the activity of fatty acid biosynthesis, we determined mRNA expression levels of acetyl-CoA carboxylases 1 and 2 (*ACC1* and *ACC2* genes) converting acetyl-CoA to malonyl-CoA, and fatty acid synthase (*FASN* gene) catalyzing synthesis of palmitate from acetyl-CoA and malonyl-CoA. Both *ACC1* and *ACC2* genes are required for fatty acid biosynthesis and inhibit fatty acid oxidation. In particular, malonyl-CoA produced by ACC1 serves as a substrate for fatty acid biosynthesis more effectively than inhibition of fatty acid oxidation, whereas malonyl-CoA produced by ACC2 serves to inhibit CPT1 more than fatty acid biosynthesis, thus preventing fatty acid oxidation^45^ (Fig. 4a). *ACC1* and *ACC2* genes were higher expressed in LCSCs than non-LCSCs, but *FASN* gene appeared to be slightly decreased in LCSCs (Fig. 4a and f); all three genes catalyze fatty acid biosynthesis. It should be noted that *ACC2*, significantly more expressed in LCSCs, also indirectly downregulates fatty acid oxidation. This result also supports our observation that fatty acid oxidation is likely to be less active in LCSCs. Taken together, fatty acid biosynthesis is likely to be not changed between LCSCs and non-LCSCs. Uncertainties that led to the lack of statistical significances for some results could be attributed to incomplete cell sorting (LCSCs *vs* non-LCSCs), different cell conditions and possibly less differences in fatty acid metabolism activities between LCSCs and non-LCSCs in reality.

As we characterized the metabolism of LCSCs, understanding metabolism of LCSCs is important for developing therapeutic strategies, such as targeted therapy, and discovering potential cures for the resistance of liver cancer cells to anticancer treatments. Based on integrative analysis results, we observed that LCSCs have more active glycolysis and less active fatty acid oxidation compared with non-LCSCs. In particular, aerobic glycolysis has important roles in cancer cell resistance. However, it is not clear that aerobic glycolysis in liver cancer could be used as a potential target for therapy, and this possibility remains the most intriguing. Our systems approach may be useful for understanding the biological significance of aerobic glycolysis in liver cancer. In addition, we also suggested that MYC and SIRT1 play important roles in reprogrammed metabolism in LCSCs. For instance, MYC and SIRT1 synergistically promote proliferation of liver cancer cells and predict a poor prognosis of HCC patients^46^.

In summary, we systematically analyzed and compared the global metabolic phenotype of LCSCs using transcriptome, metabolome and GEMs and compared this with the metabolism of non-LCSCs. We first identified that LCSCs have more rapid proliferation compared with non-LCSCs. We also suggested that more rapid cell proliferation of LCSCs can be achieved by rewired global transcriptional and metabolic changes. Along with experimental validation of the expression of key metabolic genes and signaling factors, we suggest that LCSCs exhibit less active fatty acid oxidation compared with non-LCSCs. Moreover, LCSCs are likely to depend more on glycolytic ATP production rather than non-LCSCs, whereas ATP production by oxidative phosphorylation induced by fatty acid oxidation is inhibited by increased expression of the *ACC2* gene and acetylated PGC-1α. The present study is the first to demonstrate global metabolic characteristics of LCSCs, and the insights gleaned from this study will be helpful to develop therapeutic strategies for HCC.

## Materials and Methods

### Cell culture and flow cytometric analysis

The human hepatoma Huh7 cell line was obtained from the Human Science Research Resources Bank (Tokyo, Japan). Cells were grown in Dulbecco’s modified Eagle’s medium (DMEM; Invitrogen, Carlsbad, CA, USA) supplemented with 10% fetal bovine serum (FBS; Invitrogen), 100 μg/ml penicillin, and 0.25 μg/ml streptomycin and maintained in a humidified 37 °C incubator with 5% CO_2_. Cells were harvested with 0.5 mM trypsin/EDTA (Invitrogen) and subsequently incubated at 4 °C with a phycoerythrin (PE)-conjugated anti-CD133/1 antibody (Miltenyi Biotec, Auburn, CA). LCSCs were sorted from Huh7 cells using flow cytometry (MoFlo XDP: Beckman Coulter, Miami, FL, USA) with an antibody against CD133/1. Isotype-matched mouse IgG was used as a control.

### Sphere formation assay

Cells were plated in ultra-low attachment multiwell plates (Corning Costar Corp., Cambridge, MA, USA) at a density of 2 × 10^2^ cells per well in serum-free DMEM/F12 (Invitrogen) with B27 supplement (Invitrogen), basic fibroblast growth factor (bFGF, 20 ng/ml; PeproTech, Rocky Hill, NJ, USA) and epidermal growth factor (EGF, 20 ng/ml; PeproTech). Cells were incubated at 37 °C in an atmosphere of 5% CO_2_ for 5 days. Subsequently, the number of spheres (diameter > 50 um) in each well was counted using an inverted microscope (Olympus, Tokyo, Japan). The average number of spheres was calculated from three independent experiments.

### Cell proliferation assay

Proliferation assays were conducted in 6-well plates, starting with a cell density of 10^4 ^cells/cm^2^. At the time points indicated in Fig. 1e, LCSCs and non-LCSCs were trypsinized, collected by centrifugation, and resuspended in PBS. The total number of cells in each sample was determined by hemocytometer counting, and the ratio of the final cell number to the initial number of plated cells was determined.

### Tumor xenograft model

All animal experiments were performed in accordance with institutional guidelines and were approved by the Institutional Animal Care and Use Committee of The Catholic University of Korea. Five-week-old Balb/c nude male mice (Central Lab. Animal Inc., Seoul, Korea) were housed in the animal facility for least 2 weeks before starting the experiments. In order to establish a subcutaneous xenograft model, sorted LCSCs and non-LCSCs were resuspended in FBS-free culture medium and subcutaneously injected into the left and right legs of mice at 2 × 10^6^ cells/mouse. Tumor size was measured using calipers, and tumor volume was calculated as (length × width^2^)/2. The average volume at each time point was plotted (n = 3; *P < 0.05 and **P < 0.001 for LCSC *vs* non-LCSC).

### Transcriptome data analysis

Transcriptome data were obtained from our previous study^16^ and include duplicate gene expression profiles of the two cell types; LCSCs and non-LCSCs treated without irradiation. The data are available at the Gene Expression Omnibus (GEO accession number: GSE22247). Transcriptome analysis for the differential expression was conducted using Expander 7.1^47^. Genes with P < 0.05 by Student’s t-tests were considered as differentially expressed genes.

### Functional enrichment analysis

Functional enrichment analysis was conducted by mapping genes with significant expression level changes onto the Gene Ontology (GO) biological process terms using the Database for Annotation, Visualization, and Integrated Discovery (DAVID)^48^. Transcriptome data from LCSCs and non-LCSCs treated without irradiation were used for the functional enrichment analysis. Significantly up- and down-regulated genes in LCSCs were used as input genes for DAVID to determine their involvement in the biological processes. A P < 0.01 was considered as a threshold to determine functional characteristics.

### Metabolome data analysis

Metabolome analysis was performed in samples of sorted cells according to the protocol provided by Human Metabolome Technologies, Inc. (HMT, Yamagata, Japan). The filtrate was concentrated by centrifugation and dissolved with Milli-Q water immediately before the measurement. The compounds were measured in the Cationic and Anionic modes of Capillary Electrophoresis time-of flight mass spectrometry (CE-TOFMS)-based metabolome analysis. The samples were diluted 2-fold and 10-fold for the measurement of cation and anion modes, respectively, to improve sensitivity of the CE-MS analysis.

Peaks detected in CE-TOFMS analysis were extracted using automatic integration software (MasterHands ver. 2.16.0.15 developed at Keio University) to obtain peak information including m/z, migration time (MT), and peak area. All target metabolites were then assigned from HMT’s standard library and Known-Unknown peak library on the basis of m/z and MT. The tolerance was ±0.5 min in MT and ±10 ppm in m/z.

### Reconstruction of LCSC and non-LCSC GEMs

The generic human metabolic model HMR 2.0 was used as a template model^27^. The tINIT algorithm was applied to the HMR 2.0, which is an omics integration method maximizing the consistency of omics data by solving mixed integer programming problem (MILP), to generate LCSC and non-LCSC GEMs^28^. Three input datasets were prepared for implementation of the tINIT. First, gene expression values of transcriptome data were transformed to their rank values because hierarchical clustering of the transcriptome data with rank values produced clearer patterns than the transcriptome data with expression values (see Fig. S2). Among all the genes from the transcriptome data, only metabolic genes present in the HMR 2.0 model were considered for this study. All the considered genes were sorted in descending order based on their expression levels, and were given rank values accordingly. A gene with the greatest expression level receives the highest rank value (i.e., the total number of genes), and a gene with the lowest expression level receives the lowest rank value (i.e., ‘1’). The score of each gene was assigned by adjusting their rank values (i.e., dividing the rank value of each gene with a total number of genes * 0.3 where 0.3 represents bottom 30% of genes in the rank). Second, among all the detected standard and putative metabolites, 82 and 78 corresponding metabolites present in HMR 2.0 were set to be produced in the LCSC and non-LCSC GEMs, respectively, to ensure that the corresponding metabolites are intracellularly produced (Supplementary Table S4). Finally, 56 essential metabolic tasks that occur in human cells regardless of the cell type (e.g., *de novo* biosynthesis of nucleotides, uptake of essential amino acids and fatty acid oxidation) were considered in the tINIT algorithm to ensure the correct model functionality. With the three input data, the tINIT algorithm was implemented using the RAVEN Toolbox^49^ under MATLAB R2013a (MathWorks Inc., Natick, MA) environment. After the reconstruction, the GrowMatch algorithm was performed to enable the cell type-specific GEMs to demonstrate growth under RPMI-1640 medium by adding a minimum number of reactions from the HMR 2.0 to the LCSC and non-LCSC GEMs (Supplementary Table S5)^50^,^51^. The GrowMatch algorithm was implemented under Python environment with Gurobi Optimizer 6.0 and *GurobiPy* package (Gurobi Optimization, Inc., Houston, TX).

### Prediction of transcription factors responsible for LCSCs proliferation

Biomass reaction of each LCSC GEM was forced to gradually increase its flux value from 90% to 100% of growth rate. Simultaneously, flux range (i.e., minimum and maximum) of each reaction was calculated at each step. After calculation, Pearson correlation coefficient between each reaction and biomass reaction was calculated. In this stage, we used mean flux of each reaction. Reactions with Pearson correlation coefficient >0.7 were considered as growth associated reactions. Metabolic genes mediating these reactions were obtained from gene-protein-reaction (GPR) associations defined in the LCSC GEMs. Information on transcription factors and their target genes was obtained from RegNetwork^31^. We downloaded high confidence data with experimental evidence. These metabolic simulations were performed in the Python environment with Gurobi Optimizer 6.0 and *GurobiPy* package (Gurobi Optimization, Inc., Houston, TX). Pearson correlation coefficient was calculated using python package *SciPy*^52^. Reading and writing of the SBML models were implemented using *COBRApy*^51^ and the RAVEN Toolbox^49^.

### Quantitative real-time reverse transcriptase-polymerase chain reaction (qRT-PCR)

Total RNA was extracted using TRIzol reagent (Invitrogen) according to the manufacturer’s protocol. Complementary DNA (cDNA) was synthesized from 1 μg of total RNA using reverse transcriptase (Promega, Madison, WI) and random primers (Promega) and amplified using Lightcycler 480 Probes Master real-time PCR master mix (Roche Applied Science, Indianapolis, IN) in combination with Universal Probe Library (UPL) assays (Roche Applied Science). Assays were designed according to publicly available gene sequences (NCBI) using ProbeFinder UPL software (v.2.45) (Roche Applied Science). Each 20 μL PCR reaction comprised 0.4 μM target primers, 0.4 μM target UPL, 0.4 μM reference primers, 0.4 μM reference probe, and Roche real-time PCR master mix. The cycling conditions were as follows: preincubation at 95 °C for 10 min, followed by 45 cycles at 95 °C for 10 s, 55 °C for 45 s, and 72 °C for 1 s. Human β-actin was used as reference genes. All fluorescence data were analyzed using LightCycler 4.0 software (Roche Applied Science), and Ct results were exported to Excel (Microsoft, Redmond, WA). Gene expression was quantified and normalized using the comparative Ct method.

### PGC-1α acetylation assay and western blot analysis

PGC-1α lysine acetylation was analyzed by immunoprecipitation of PGC-1α followed by Western blot using anti-acetyl-lysine antibodies (Cell Signaling Technology, Beverly, MA, USA). Protein extracts were obtained as described^53^. PGC-1α levels and acetylation were detected using specific antibodies for PGC-1α and acetyl-lysine.

The immunoprecipitates and protein extracts were separated by SDS-polyacrylamide gel electrophoresis, transferred to nitrocellulose membranes (Schleicher & Schuell, Dassel, Germany) and blocked in 5% skim milk. Primary antibodies were used as indicated by the manufacturer and include the following: SIRT1, PGC-1α, 14-3-3δ (Santa Cruz Biotechnology, Santa Cruz, CA, USA) and β-actin (Sigma-Aldrich, St. Louis, MO, USA).

## Additional Information

**How to cite this article:** Hur, W. *et al*. Systems approach to characterize the metabolism of liver cancer stem cells expressing CD133. *Sci. Rep.* **7**, 45557; doi: 10.1038/srep45557 (2017).

**Publisher's note:** Springer Nature remains neutral with regard to jurisdictional claims in published maps and institutional affiliations.

## Supplementary Material

Supplementary Information
