# Supplementary material for: Systems approach to characterize the metabolism of liver cancer stem cells expressing CD133
Source: Sci Rep. 2017 Apr 3;7:45557. doi: 10.1038/srep45557 (PMC5377334; doi:10.1038/srep45557)
Supplement: Supplementary Information [file srep45557-s1.doc]

**Supplementary Information**

**Systems approach to characterize the metabolism of liver cancer stem cells expressing CD133**

Wonhee Hur, Jae Yong Ryu, Hyun Uk Kim, Sung Woo Hong, Eun Byul Lee, Sang Yup Lee and Seung Kew Yoon


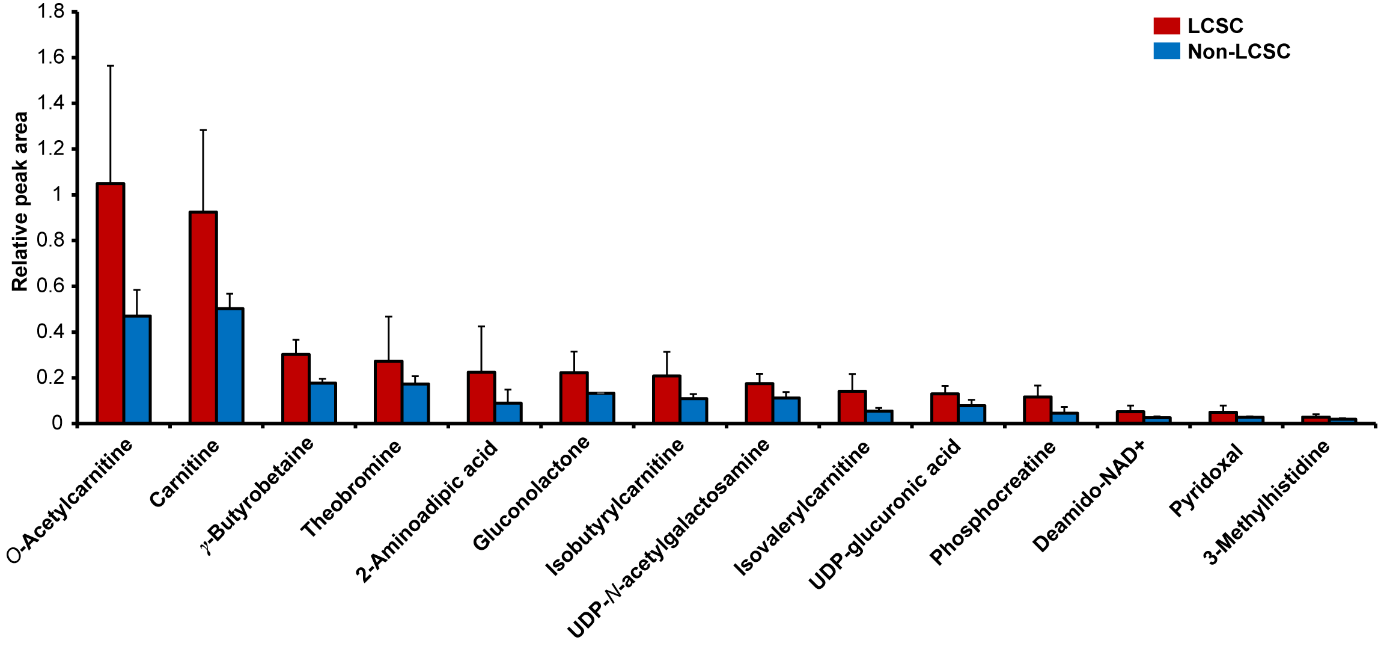


**Supplementary Figure S1.** **Comparison of relative abundances of putative metabolites.** Relative concentrations of putative metabolites with more than 1.5-fold increased abundance in LCSCs (red bars), compared to non-LCSCs (blue bars). All the data samples were in duplicates. Error bars indicate mean ± S.D.


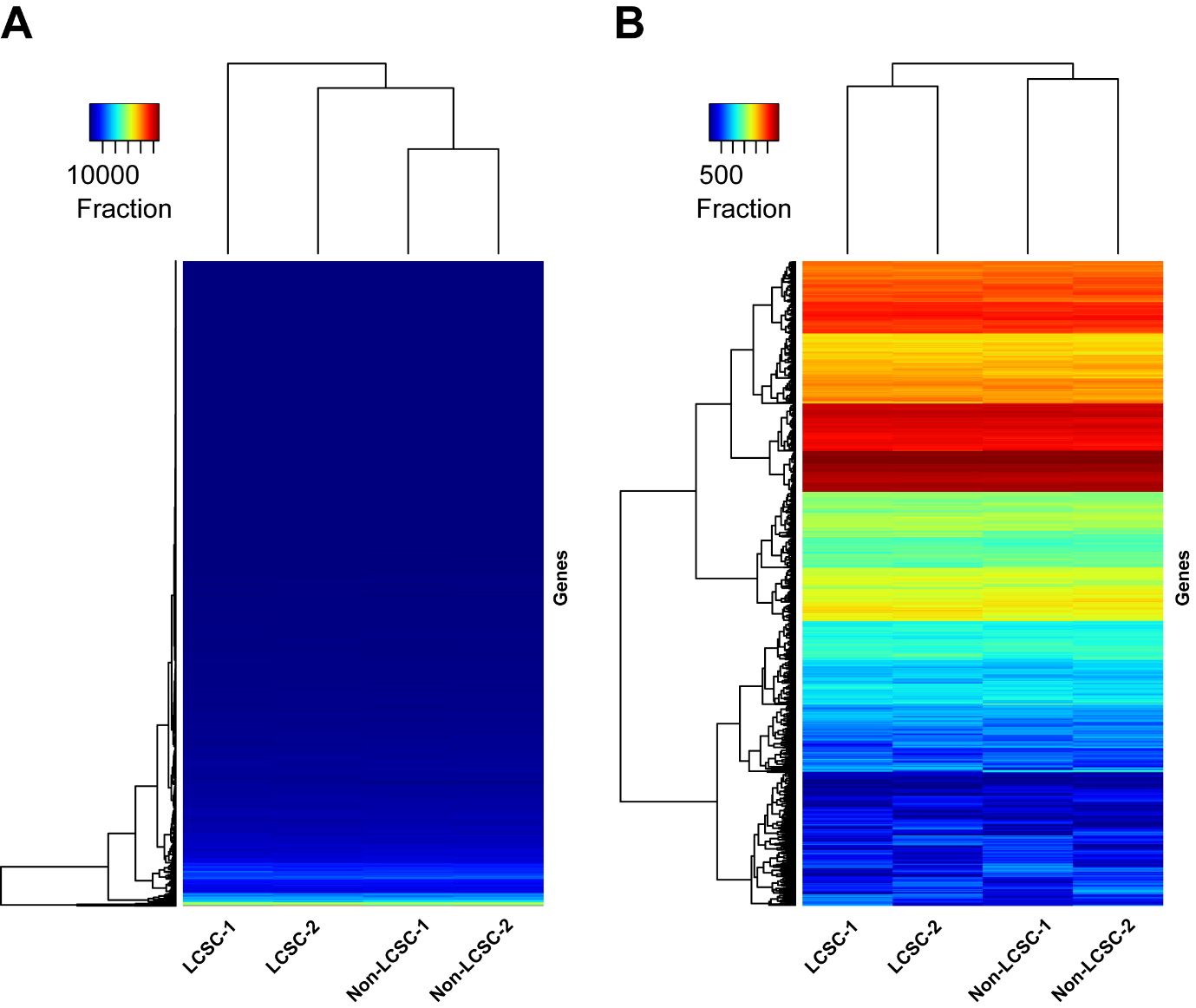


**Supplementary Figure S2.** **Comparisons between absolute expression values and rank values.** **A.** Heat map for expression patterns between LCSCs and non-LCSCs using absolute expression values. **B.** Heat map for expression patterns between LCSCs and non-LCSCs using rank values which are transformed by absolute expression values.

**Supplementary Table S1. Gene ontology (GO) biological process enrichment analysis for differentially expressed genes between LCSCs and non-LCSCs.**

| **Group** | **GO ID** | **GO term** | **P-value** | **Gene symbols** |
| --- | --- | --- | --- | --- |
| Genes up-regulated in  LCSCs | GO:0050818 | regulation of coagulation | 0.007822382 | *EDN1, F2RL1, ANXA4* |
|  | GO:0009611 | response to wounding | 0.027966858 | *CD55, FBLN5, AOX1, F2RL1, MGLL, CFD* |
|  | GO:0030335 | positive regulation of cell migration | 0.033863794 | *EDN1, F2RL1, LAMB1* |
|  | GO:0006865 | amino acid transport | 0.034563048 | *SLC3A1, SLC1A1, SLC7A7* |
|  | GO:0007586 | digestion | 0.035267949 | *CTSE, TFF2, TFF3* |
|  | GO:0051272 | positive regulation of cell motion | 0.04035684 | *EDN1, F2RL1, LAMB1* |
|  | GO:0040017 | positive regulation of locomotion | 0.04035684 | *EDN1, F2RL1, LAMB1* |
|  | GO:0042127 | regulation of cell proliferation | 0.040726615 | *CAV2, CDKN1A, CXCL5, EDN1, GUCY2C, LAMB1, TIMP2* |
|  | GO:0002684 | positive regulation of immune system process | 0.041987752 | *CDKN1A, CD55, F2RL1, CFD* |
|  | GO:0030198 | extracellular matrix organization | 0.044926552 | *LGALS3, FBLN5, COL2A1* |
|  | GO:0007204 | elevation of cytosolic calcium ion concentration | 0.04967891 | *CD55, EDN1, F2RL1* |
| Genes down-regulated in  LCSCs | GO:0007389 | pattern specification process | 0.024734218 | *NRP1, CYP26B1, RELN, LFNG* |
|  | GO:0042445 | hormone metabolic process | 0.025891998 | *SCPEP1, CYP26B1, RELN* |
|  | GO:0042573 | retinoic acid metabolic process | 0.028030419 | *SCPEP1, CYP26B1* |
|  | GO:0006508 | proteolysis | 0.034744515 | *SCPEP1, PGC, LGMN, CFH, CUL4B, CPB2, NSF* |
|  | GO:0009611 | response to wounding | 0.035160145 | *A2M, NRP1, CFH, LYZ, CD302* |
|  | GO:0035094 | response to nicotine | 0.039483755 | *LEPR, CHRNA5* |
|  | GO:0006954 | inflammatory response | 0.040779008 | *A2M, CFH, LYZ, CD302* |
|  | GO:0010817 | regulation of hormone levels | 0.049353192 | *SCPEP1, CYP26B1, RELN* |
|  | GO:0006776 | vitamin A metabolic process | 0.05305524 | *SCPEP1, CYP26B1* |
|  | GO:0001523 | retinoid metabolic process | 0.05305524 | *SCPEP1, CYP26B1* |
|  | GO:0016101 | diterpenoid metabolic process | 0.05305524 | *SCPEP1, CYP26B1* |
|  | GO:0032101 | regulation of response to external stimulus | 0.054108773 | *AGTR1, A2M, CPB2* |
|  | GO:0006721 | terpenoid metabolic process | 0.057537647 | *SCPEP1, CYP26B1* |
|  | GO:0006775 | fat-soluble vitamin metabolic process | 0.075262607 | *SCPEP1, CYP26B1* |
|  | GO:0003002 | regionalization | 0.078724208 | *CYP26B1, RELN, LFNG* |
|  | GO:0031667 | response to nutrient levels | 0.078724208 | *A2M, LEPR, CP* |
|  | GO:0009991 | response to extracellular stimulus | 0.095038086 | *A2M, LEPR, CP* |
|  | GO:0006720 | isoprenoid metabolic process | 0.099107989 | *SCPEP1, CYP26B1* |

**Supplementary Table S2. Concentrations of standard metabolites (pmol/10^6^ cells).**

| **Metabolite name** | **LCSC-1** | **LCSC-2** | **Non-LCSC-1** | **Non-LCSC-2** |
| --- | --- | --- | --- | --- |
| Adenosine | 79.303 | 170.520 | 57.752 | 84.912 |
| ADP | 3145.696 | 5100.465 | 2231.740 | 3735.686 |
| Ala | 2507.987 | 45194.392 | 2708.009 | 25488.214 |
| Arg | 417.177 | 1329.826 | 406.475 | 1068.207 |
| Asp | 1763.358 | 11515.025 | 1414.060 | 4927.628 |
| ATP | 15891.290 | 37069.873 | 10401.822 | 20179.352 |
| Betaine | 1481.093 | 2937.221 | 1609.926 | 2038.455 |
| Choline | 7777.612 | 8455.293 | 6054.079 | 5736.106 |
| Citric acid | 9124.359 | 14114.647 | 5255.575 | 7821.660 |
| Citrulline | 205.911 | 1285.807 | 412.252 | 798.827 |
| Creatine | 4035.481 | 14337.162 | 4416.268 | 7188.770 |
| Creatinine | 256.334 | 499.889 | 296.264 | 347.107 |
| CTP | 2114.210 | 3532.238 | 1435.646 | 2183.886 |
| Dihydroxyacetone phosphate | 6763.786 | 9599.742 | 5842.772 | 8135.158 |
| Fructose 1,6-diphosphate | 2284.738 | 3122.841 | 1547.116 | 4010.246 |
| GABA | 613.517 | 1911.802 | 1045.123 | 1859.089 |
| Gln | 6739.397 | 71072.799 | 6662.155 | 46671.964 |
| Glu | 22500.291 | 341956.984 | 21721.484 | 168855.230 |
| Gluconic acid | 3025.212 | 4033.515 | 1298.549 | 1584.636 |
| Glucose 1-phosphate | 685.265 | 823.715 | 605.294 | 745.389 |
| Glucose 6-phosphate | 3308.579 | 3761.361 | 2488.001 | 2951.267 |
| Glutathione (GSH) | 305.266 | 2371.269 | 307.658 | 1954.394 |
| Glutathione (GSSG)_divalent | 15656.389 | 92178.494 | 15683.025 | 49220.309 |
| Gly | 14762.666 | 54409.043 | 17797.284 | 43736.340 |
| Glyceraldehyde 3-phosphate | 2308.541 | 2271.489 | 2534.241 | 2045.788 |
| Glycerol 3-phosphate | 3907.945 | 5369.005 | 2724.686 | 3510.223 |
| GTP | 4963.655 | 8332.348 | 3241.084 | 5290.189 |
| His | 2791.231 | 5250.642 | 3009.287 | 3913.303 |
| Hydroxyproline | 389.289 | 1256.576 | 479.702 | 766.116 |
| Ile | 499.339 | 11761.177 | 543.257 | 6611.869 |
| Lactic acid | 91590.081 | 332043.678 | 79268.672 | 169858.275 |
| Leu | 573.605 | 10881.883 | 694.982 | 6141.725 |
| Lys | 786.620 | 3356.023 | 874.882 | 2894.246 |
| Malic acid | 19504.551 | 24880.135 | 13615.710 | 18813.422 |
| NAD+ | 6511.213 | 11988.190 | 4675.911 | 6683.583 |
| Ornithine | 80.334 | 429.531 | 121.557 | 351.867 |
| Phe | 667.813 | 6901.715 | 696.449 | 3793.339 |
| Pro | 11150.746 | 51418.219 | 15062.047 | 30103.452 |
| S-Adenosylmethionine | 325.917 | 1497.348 | 309.937 | 1023.601 |
| Ser | 4535.487 | 16167.785 | 3744.147 | 13003.568 |
| Spermidine | 357.126 | 450.618 | 439.634 | 485.238 |
| Succinic acid | 8955.841 | 14780.109 | 6533.439 | 8867.661 |
| Thr | 14501.969 | 39837.109 | 13589.933 | 24547.919 |
| Trp | 61.879 | 314.041 | 82.041 | 215.727 |
| Tyr | 903.540 | 7306.974 | 939.474 | 4011.705 |
| UTP | 5185.171 | 9015.692 | 3476.600 | 5474.051 |
| Val | 639.617 | 14900.684 | 729.623 | 8310.713 |
| β-Ala | 8445.726 | 21109.836 | 10130.597 | 16979.757 |
| 2-Hydroxybutyric acid | ND | ND | ND | ND |
| 2-Oxoglutaric acid | ND | ND | ND | ND |
| 2-Oxoisovaleric acid | ND | ND | ND | ND |
| 2-Phosphoglyceric acid | ND | ND | ND | ND |
| 3-Hydroxybutyric acid | ND | 880.882 | ND | 624.884 |
| 3-Phosphoglyceric acid | ND | ND | ND | ND |
| 6-Phosphogluconic acid | ND | ND | ND | ND |
| Acetyl CoA_divalent | ND | ND | ND | ND |
| Adenine | ND | ND | ND | 67.925 |
| AMP | 726.159 | 1104.380 | ND | 1026.355 |
| Anthranilic acid | ND | ND | ND | ND |
| Asn | ND | 654.487 | ND | 423.233 |
| Betaine aldehyde_+H2O | ND | 145.591 | ND | 498.152 |
| cAMP | ND | ND | ND | ND |
| Carnosine | ND | 178.884 | ND | 57.601 |
| CDP | ND | ND | ND | ND |
| cGMP | ND | ND | ND | ND |
| cis-Aconitic acid | ND | ND | ND | ND |
| CMP | ND | ND | ND | ND |
| CoA_divalent | ND | ND | ND | ND |
| Cys | ND | ND | ND | ND |
| Cytidine | ND | ND | ND | ND |
| Cytosine | ND | ND | ND | ND |
| dATP | ND | ND | ND | ND |
| dCTP | ND | ND | ND | ND |
| dTDP | ND | ND | ND | ND |
| dTMP | ND | ND | ND | ND |
| dTTP | ND | ND | ND | ND |
| Erythrose 4-phosphate | ND | ND | ND | ND |
| Fructose 6-phosphate | ND | ND | ND | ND |
| Fumaric acid | ND | ND | ND | ND |
| GDP | ND | 888.455 | ND | ND |
| Glycolic acid | ND | ND | ND | ND |
| Glyoxylic acid | ND | ND | ND | ND |
| GMP | ND | ND | ND | ND |
| Guanine | ND | ND | ND | ND |
| Guanosine | ND | ND | ND | ND |
| Homoserine | ND | ND | ND | ND |
| Hypoxanthine | ND | ND | ND | ND |
| IMP | ND | ND | ND | ND |
| Inosine | ND | ND | ND | ND |
| Isocitric acid | ND | ND | ND | ND |
| Malonyl CoA_divalent | ND | ND | ND | ND |
| Met | ND | ND | ND | ND |
| N,N-Dimethylglycine | ND | ND | ND | ND |
| NADP+ | ND | 899.859 | ND | ND |
| Phosphoenolpyruvic acid | ND | ND | ND | ND |
| PRPP | ND | ND | ND | ND |
| Putrescine | ND | ND | ND | ND |
| Pyruvic acid | ND | ND | ND | ND |
| Ribose 5-phosphate | ND | ND | ND | ND |
| Ribulose 5-phosphate | ND | ND | ND | ND |
| Sarcosine | ND | 223.681 | ND | ND |
| Sedoheptulose 7-phosphate | ND | ND | ND | ND |
| Spermine | ND | 1484.695 | ND | 481.468 |
| Thymidine | ND | ND | ND | ND |
| Thymine | ND | ND | ND | ND |
| Tyramine | ND | ND | ND | ND |
| UDP | ND | 938.961 | ND | ND |
| UMP | ND | ND | ND | ND |
| Uracil | ND | ND | ND | ND |
| Uridine | ND | ND | ND | ND |

ND, not detected.

**Supplementary Table S3. Relative peaks area of putative metabolites.**

| **Metabolite** | **LCSC-1** | **LCSC-2** | **Non-LCSC-1** | **Non-LCSC-2** |
| --- | --- | --- | --- | --- |
| Isovalerylcarnitine | 0.065 | 0.217 | 0.041 | 0.069 |
| Phosphocreatine | 0.066 | 0.167 | 0.018 | 0.073 |
| 2-Aminoadipic acid | 0.024 | 0.425 | 0.030 | 0.148 |
| Gluconic acid | 0.158 | 0.210 | 0.068 | 0.083 |
| O-Acetylcarnitine | 0.533 | 1.564 | 0.355 | 0.584 |
| Asp | 0.264 | 1.722 | 0.211 | 0.737 |
| Deamido-NAD+ | 0.024 | 0.079 | 0.019 | 0.032 |
| Glu | 4.138 | 62.893 | 3.995 | 31.056 |
| Isobutyrylcarnitine | 0.102 | 0.314 | 0.089 | 0.129 |
| Carnitine | 0.565 | 1.283 | 0.437 | 0.568 |
| Pyridoxal | 0.020 | 0.079 | 0.023 | 0.031 |
| Citric acid | 0.594 | 0.919 | 0.342 | 0.509 |
| Adenosine | 0.020 | 0.044 | 0.015 | 0.022 |
| ATP | 0.410 | 0.956 | 0.268 | 0.520 |
| Val | 0.255 | 5.940 | 0.291 | 3.313 |
| XC0061 | 0.434 | 1.221 | 0.430 | 0.534 |
| Ile | 0.253 | 5.970 | 0.276 | 3.356 |
| γ-Butyrobetaine | 0.238 | 0.366 | 0.158 | 0.196 |
| Lactic acid | 2.953 | 10.707 | 2.556 | 5.477 |
| Ala | 0.527 | 9.496 | 0.569 | 5.355 |
| Phe | 0.292 | 3.018 | 0.305 | 1.659 |
| Gluconolactone | 0.129 | 0.315 | 0.131 | 0.133 |
| Leu | 0.314 | 5.962 | 0.381 | 3.365 |
| Glutathione (GSSG)_divalent | 3.426 | 20.172 | 3.432 | 10.771 |
| Tyr | 0.205 | 1.661 | 0.214 | 0.912 |
| UDP-glucuronic acid | 0.095 | 0.165 | 0.053 | 0.103 |
| NAD+ | 0.094 | 0.172 | 0.067 | 0.096 |
| XC0016 | 0.111 | 0.534 | 0.125 | 0.280 |
| UTP | 0.146 | 0.255 | 0.098 | 0.155 |
| Creatine | 1.121 | 3.984 | 1.227 | 1.998 |
| Theobromine | 0.078 | 0.468 | 0.138 | 0.207 |
| UDP-N-Acetylgalactosamine UDP-N-Acetylglucosamine | 0.131 | 0.218 | 0.085 | 0.138 |
| CTP | 0.037 | 0.061 | 0.025 | 0.038 |
| GTP | 0.088 | 0.148 | 0.058 | 0.094 |
| 3-Methylhistidine | 0.017 | 0.041 | 0.013 | 0.024 |
| Succinic acid | 0.453 | 0.747 | 0.330 | 0.448 |
| Glycerol 3-phosphate | 0.133 | 0.182 | 0.093 | 0.119 |
| Gln | 1.244 | 13.122 | 1.230 | 8.617 |
| 5-Oxoproline | 0.275 | 0.782 | 0.238 | 0.488 |
| Thiamine | 0.098 | 0.061 | 0.065 | 0.044 |
| Butyrylcarnitine | 0.045 | 0.110 | 0.039 | 0.069 |
| 4-Acetamidobutanoic acid | 0.289 | 0.171 | 0.201 | 0.121 |
| UDP-Glucose UDP-Galactose | 0.130 | 0.210 | 0.100 | 0.138 |
| Thr | 2.847 | 7.822 | 2.668 | 4.820 |
| N-Acetylglycine | 0.285 | 0.214 | 0.203 | 0.150 |
| Pro | 3.299 | 15.211 | 4.456 | 8.905 |
| ADP | 0.089 | 0.145 | 0.063 | 0.106 |
| Choline | 3.390 | 3.686 | 2.639 | 2.500 |
| Malic acid | 1.449 | 1.849 | 1.012 | 1.398 |
| S-Adenosylmethionine | 0.034 | 0.156 | 0.032 | 0.107 |
| Pantothenic acid | 0.192 | 0.231 | 0.143 | 0.171 |
| Cysteinesulfinic acid | 0.206 | 0.130 | 0.144 | 0.106 |
| 6-Aminohexanoic acid | 0.051 | 0.076 | 0.039 | 0.056 |
| Homocysteic acid | 1.575 | 1.017 | 1.105 | 0.845 |
| Hydroxyproline | 0.075 | 0.241 | 0.092 | 0.147 |
| Methionine sulfoxide | 0.078 | 0.640 | 0.109 | 0.442 |
| 3-Guanidinopropionic acid | 0.021 | 0.055 | 0.021 | 0.037 |
| Glucose 6-phosphate | 0.139 | 0.158 | 0.105 | 0.124 |
| Phenaceturic acid | 0.078 | 0.049 | 0.061 | 0.037 |
| Threonic acid | 0.071 | 0.159 | 0.061 | 0.118 |
| Trp | 0.018 | 0.091 | 0.024 | 0.062 |
| Pyridoxine | 0.153 | 0.292 | 0.157 | 0.197 |
| N-Acetylaspartic acid | 0.093 | 0.081 | 0.084 | 0.056 |
| 4-Guanidinobutyric acid | 0.025 | 0.076 | 0.033 | 0.048 |
| Ser | 0.758 | 2.702 | 0.626 | 2.173 |
| Citrulline | 0.041 | 0.255 | 0.082 | 0.159 |
| Betaine | 0.444 | 0.880 | 0.482 | 0.611 |
| N-Acetylasparagine | 0.225 | 0.298 | 0.203 | 0.236 |
| 2-Hydroxyglutaric acid | 0.103 | 0.146 | 0.085 | 0.123 |
| Arg | 0.122 | 0.389 | 0.119 | 0.312 |
| Glutathione (GSH) | 0.050 | 0.390 | 0.051 | 0.321 |
| Creatinine | 0.071 | 0.138 | 0.082 | 0.096 |
| Dihydroxyacetone phosphate | 0.207 | 0.294 | 0.179 | 0.249 |
| His | 0.639 | 1.202 | 0.689 | 0.896 |
| Gly | 1.904 | 7.017 | 2.295 | 5.640 |
| Glucose 1-phosphate | 0.030 | 0.036 | 0.026 | 0.032 |
| N-Formylglycine | 0.124 | 0.091 | 0.098 | 0.095 |
| Ketoprofen | 0.078 | 0.104 | 0.065 | 0.099 |
| Lys | 0.168 | 0.716 | 0.187 | 0.617 |
| β-Ala | 1.570 | 3.924 | 1.883 | 3.156 |
| N-Acetylglutamic acid | 0.026 | 0.054 | 0.026 | 0.047 |
| 2-Amino-2-(hydroxymethyl)-1,3-propanediol | 0.062 | 0.070 | 0.058 | 0.064 |
| Ornithine | 0.019 | 0.104 | 0.030 | 0.085 |
| Phosphorylcholine | 27.377 | 46.546 | 32.312 | 38.464 |
| Glycerophosphocholine | 1.078 | 3.183 | 2.039 | 2.065 |
| Terephthalic acid | 0.069 | 0.056 | 0.072 | 0.050 |
| Glyceraldehyde 3-phosphate | 0.025 | 0.024 | 0.027 | 0.022 |
| Gly-Asp | 0.033 | 0.067 | 0.044 | 0.057 |
| Taurine | 5.424 | 7.638 | 6.231 | 7.033 |
| Fructose 1,6-diphosphate | 0.069 | 0.095 | 0.047 | 0.122 |
| 3-Aminoisobutyric acid | 0.046 | 0.084 | 0.050 | 0.087 |
| Spermidine | 0.101 | 0.128 | 0.124 | 0.137 |
| GABA | 0.143 | 0.447 | 0.244 | 0.435 |
| Triethanolamine | 0.038 | 0.021 | 0.036 | 0.033 |
| Glycerol | 26.558 | 15.205 | 30.393 | 19.343 |
| N-Acetylserine | 0.120 | 0.282 | 0.205 | 0.278 |
| N-Acetylornithine | 0.056 | 0.087 | 0.088 | 0.115 |
| Diethanolamine | ND | 0.032 | 0.044 | 0.052 |
| AMP | 0.022 | 0.034 | ND | 0.032 |
| Cysteine glutathione disulfide | 0.017 | 0.120 | ND | 0.064 |
| Streptomycin sulfate_+H2O_divalent | ND | 0.014 | 0.011 | 0.013 |
| trans-Glutaconic acid | ND | 0.123 | 0.085 | 0.103 |
| Nicotinic acid | ND | 0.094 | 0.037 | 0.087 |
| Isobutylamine | 0.082 | 0.178 | ND | 0.072 |
| Betaine aldehyde_+H2O | ND | 0.040 | ND | 0.136 |
| Urocanic acid | ND | 0.016 | 0.029 | ND |
| 1-Aminocyclopropane-1-carboxylic acid Homoserinelactone | ND | 0.020 | 0.021 | ND |
| 5-Aminovaleric acid | ND | 0.138 | ND | 0.103 |
| 3-Hydroxybutyric acid | ND | 0.032 | ND | 0.023 |
| Ethanolamine | ND | 0.042 | ND | 0.028 |
| Isovaleric acid | ND | 0.066 | ND | 0.044 |
| N-Methylglutamic acid | 0.042 | ND | 0.027 | ND |
| Cystathionine | ND | 0.066 | ND | 0.032 |
| Spermine | ND | 0.135 | ND | 0.044 |
| Carnosine | ND | 0.033 | ND | 0.011 |
| 1-Methylnicotinamide | 0.032 | 0.020 | ND | ND |
| N-Acetylneuraminic acid | 0.016 | 0.017 | ND | ND |
| Sarcosine | ND | 0.048 | ND | ND |
| GDP | ND | 0.029 | ND | ND |
| N-Methylalanine | ND | ND | ND | ND |

ND, not detected.

**Supplementary Table S4. List of metabolites which should** be produced in LCSC and/or non-LCSC GEM after reconstruction.

| **Metabolites detected in LCSCs** | **Metabolites detected in non-LCSCs** |
| --- | --- |
| (R)-3-Hydroxybutanoate | (R)-3-Hydroxybutanoate |
| 1-Methylnicotinamide | 3-Sulfinoalanine |
| 3-Sulfinoalanine | 4-Acetamidobutanoate |
| 4-Acetamidobutanoate | 4-Aminobutyrate |
| 4-Aminobutyrate | Adenosine |
| Adenosine | ADP |
| ADP | Alanine |
| Alanine | AMP |
| AMP | Arginine |
| Arginine | Aspartate |
| Aspartate | ATP |
| ATP | beta-Alanine |
| beta-Alanine | Betaine |
| Betaine | Betaine_aldehyde |
| Betaine_aldehyde | Choline |
| Choline | Citrate |
| Citrate | Citrulline |
| Citrulline | Creatine |
| Creatine | Creatine-phosphate |
| Creatine-phosphate | Creatinine |
| Creatinine | CTP |
| CTP | Deamido-NAD |
| Deamido-NAD | DHAP |
| DHAP | Ethanolamine |
| Ethanolamine | Fructose-1,6-bisphosphate |
| Fructose-1,6-bisphosphate | gamma-Butyrobetaine |
| gamma-Butyrobetaine | Glucose-1-phosphate |
| GDP | Glucose-6-phosphate |
| Glucose-1-phosphate | Glutamate |
| Glucose-6-phosphate | Glutamine |
| Glutamate | Glycerol |
| Glutamine | Glycine |
| Glycerol | GSH |
| Glycine | GSSG |
| GSH | GTP |
| GSSG | Histidine |
| GTP | Isoleucine |
| Histidine | L-2-Aminoadipate |
| Isoleucine | L-3-Amino-isobutanoate |
| L-2-Aminoadipate | L-Carnitine |
| L-3-Amino-isobutanoate | L-Cystathionine |
| L-Carnitine | Leucine |
| L-Cystathionine | L-Lactate |
| Leucine | Lysine |
| L-Lactate | Malate |
| Lysine | N-Acetyl-L-aspartate |
| Malate | N-Acetyl-L-glutamate |
| N-Acetyl-L-aspartate | N-Acetylornithine |
| N-Acetyl-L-glutamate | NAD+ |
| N-Acetylneuraminate | Nicotinate |
| N-Acetylornithine | O-Acetylcarnitine |
| NAD+ | Ornithine |
| Nicotinate | Pantothenate |
| O-Acetylcarnitine | Phenylalanine |
| Ornithine | Phosphocholine |
| Pantothenate | Proline |
| Phenylalanine | Pyridoxal |
| Phosphocholine | Pyridoxine |
| Proline | SAM |
| Pyridoxal | Serine |
| Pyridoxine | sn-Glycerol-3-PC |
| SAM | sn-Glycerol-3-phosphate |
| Sarcosine | Spermidine |
| Serine | Spermine |
| sn-Glycerol-3-PC | Succinate |
| sn-Glycerol-3-phosphate | Taurine |
| Spermidine | Thiamin |
| Spermine | Threonate |
| Succinate | Threonine |
| Taurine | trans-4-Hydroxy-L-proline |
| Thiamin | Tryptophan |
| Threonate | Tyrosine |
| Threonine | UDP-Glucose |
| trans-4-Hydroxy-L-proline | UDP-Glucuronate |
| Tryptophan | UDP-N-Acetyl-D-galactosamine |
| Tyrosine | Urocanate |
| UDP-Glucose | UTP |
| UDP-Glucuronate | Valine |
| UDP-N-Acetyl-D-galactosamine |  |
| Urocanate |  |
| UTP |  |
| Valine |  |

**Supplementary Table S5. Metabolic reaction and constraints for RPMI-1640 medium.**

| **Reaction ID** | **Lower bound** | **Upper bound** |
| --- | --- | --- |
| HMR_9067 | -0.05 | 1000 |
| HMR_9066 | -0.05 | 1000 |
| HMR_9062 | -0.05 | 1000 |
| HMR_9070 | -0.05 | 1000 |
| HMR_9065 | -0.05 | 1000 |
| HMR_9071 | -0.05 | 1000 |
| HMR_9038 | -0.05 | 1000 |
| HMR_9039 | -0.05 | 1000 |
| HMR_9040 | -0.05 | 1000 |
| HMR_9041 | -0.05 | 1000 |
| HMR_9042 | -0.05 | 1000 |
| HMR_9043 | -0.05 | 1000 |
| HMR_9068 | -0.05 | 1000 |
| HMR_9069 | -0.05 | 1000 |
| HMR_9044 | -0.05 | 1000 |
| HMR_9045 | -0.05 | 1000 |
| HMR_9064 | -0.05 | 1000 |
| HMR_9046 | -0.05 | 1000 |
| HMR_9063 | -0.5 | 1000 |
| HMR_9109 | -0.005 | 1000 |
| HMR_9083 | -0.005 | 1000 |
| HMR_9145 | -0.005 | 1000 |
| HMR_9146 | -0.005 | 1000 |
| HMR_9378 | -0.005 | 1000 |
| HMR_9144 | -0.005 | 1000 |
| HMR_9143 | -0.005 | 1000 |
| HMR_9159 | -0.005 | 1000 |
| HMR_9361 | -0.005 | 1000 |
| HMR_9082 | -1000 | 1000 |
| HMR_9081 | -1000 | 1000 |
| HMR_9150 | -1000 | 1000 |
| HMR_9077 | -1000 | 1000 |
| HMR_9072 | -1000 | 1000 |
| HMR_9034 | -5 | 1000 |
| HMR_9351 | -0.05 | 1000 |
| HMR_9048 | -1000 | 1000 |
| HMR_9047 | -1000 | 1000 |
| HMR_9076 | -1000 | 1000 |
| HMR_9061 | -0.05 | 1000 |
| HMR_9058 | -1000 | 1000 |
| HMR_9096 | -1000 | 1000 |
| HMR_9079 | -1000 | 1000 |
| HMR_9073 | -1000 | 1000 |
| HMR_9080 | -1000 | 1000 |

**Supplementary Table S6. List of metabolic pathways involved in exclusively present reactions in LCSC or non-LCSC GEMs.**

| **Group** | **Pathway** | **Number of reactions** |
| --- | --- | --- |
| **LCSC GEMs** | Transport, extracellular | 24 |
|  | Transport, lysosomal | 22 |
|  | Phenylalanine, tyrosine and tryptophan biosynthesis | 21 |
|  | Exchange reactions | 14 |
|  | Beta oxidation of di-unsaturated fatty acids (n-6) (peroxisomal) | 13 |
|  | Omega-6 fatty acid metabolism | 12 |
|  | Formation and hydrolysis of cholesterol esters | 12 |
|  | Arginine and proline metabolism | 8 |
|  | Vitamin E metabolism | 7 |
|  | Keratan sulfate degradation | 7 |
|  | Bile acid biosynthesis | 6 |
|  | Transport, mitochondrial | 5 |
|  | Arachidonic acid metabolism | 5 |
|  | Beta oxidation of even-chain fatty acids (mitochondrial) | 5 |
|  | Tricarboxylic acid cycle and glyoxylate/dicarboxylate metabolism | 4 |
|  | Transport, peroxisomal | 4 |
|  | Sphingolipid metabolism | 4 |
|  | Beta oxidation of unsaturated fatty acids (n-7) (mitochondrial) | 4 |
|  | Lysine metabolism | 4 |
|  | Vitamin D metabolism | 3 |
|  | Steroid metabolism | 3 |
|  | Fatty acid elongation (even-chain) | 3 |
|  | Fatty acid desaturation (even-chain) | 3 |
|  | Retinol metabolism | 3 |
|  | Fatty acid biosynthesis (unsaturated) | 3 |
|  | Purine metabolism | 2 |
|  | Estrogen metabolism | 2 |
|  | Alanine, aspartate and glutamate metabolism | 2 |
|  | Tryptophan metabolism | 2 |
|  | Pool reactions | 2 |
|  | Inositol phosphate metabolism | 2 |
|  | Nicotinate and nicotinamide metabolism | 2 |
|  | Glycerolipid metabolism | 2 |
|  | Glycolysis / Gluconeogenesis | 2 |
|  | Glycerophospholipid metabolism | 2 |
|  | Linoleate metabolism | 2 |
|  | Acyl-CoA hydrolysis | 2 |
|  | Glycine, serine and threonine metabolism | 2 |
|  | Pyrimidine metabolism | 2 |
|  | ROS detoxification | 2 |
|  | Fatty acid transfer reactions | 1 |
|  | Protein degradation | 1 |
|  | Pentose phosphate pathway | 1 |
|  | Glycosphingolipid metabolism | 1 |
|  | Eicosanoid metabolism | 1 |
|  | Serotonin and melatonin biosynthesis | 1 |
|  | Nucleotide metabolism | 1 |
|  | Transport, lysosome to ER | 1 |
|  | Glutathione metabolism | 1 |
|  | Cholesterol metabolism | 1 |
|  | Thiamine metabolism | 1 |
|  | Ascorbate and aldarate metabolism | 1 |
|  | Starch and sucrose metabolism | 1 |
|  | Cholesterol biosynthesis 2 | 1 |
|  | Valine, leucine, and isoleucine metabolism | 1 |
|  | prostaglandin biosynthesis | 1 |
|  | Propanoate metabolism | 1 |
|  | Fructose and Mannose metabolism | 1 |
|  | Omega-3 fatty acid metabolism | 1 |
| **Non-LCSC GEMs** | Keratan sulfate biosynthesis | 43 |
|  | N-glycan metabolism | 37 |
|  | Chondroitin / heparan sulfate biosynthesis | 31 |
|  | Transport, extracellular | 31 |
|  | Exchange reactions | 27 |
|  | Bile acid biosynthesis | 22 |
|  | Chondroitin sulfate degradation | 20 |
|  | Metabolism of xenobiotics by cytochrome P450 | 18 |
|  | Beta oxidation of poly-unsaturated fatty acids (mitochondrial) | 17 |
|  | Heparan sulfate degradation | 16 |
|  | Glycosylphosphatidylinositol (GPI)-anchor biosynthesis | 13 |
|  | Cholesterol biosynthesis 1 (Bloch pathway) | 13 |
|  | Transport, lysosomal | 12 |
|  | Leukotriene metabolism | 11 |
|  | Transport, endoplasmic reticular | 11 |
|  | Transport, mitochondrial | 11 |
|  | Fatty acid biosynthesis (unsaturated) | 9 |
|  | Vitamin E metabolism | 8 |
|  | Glycosphingolipid biosynthesis-lacto and neolacto series | 8 |
|  | Beta oxidation of unsaturated fatty acids (n-9) (peroxisomal) | 8 |
|  | Valine, leucine, and isoleucine metabolism | 8 |
|  | Beta oxidation of unsaturated fatty acids (n-7) (mitochondrial) | 8 |
|  | Transport, peroxisomal | 7 |
|  | Inositol phosphate metabolism | 7 |
|  | Keratan sulfate degradation | 7 |
|  | Retinol metabolism | 7 |
|  | Acylglycerides metabolism | 7 |
|  | Glycerophospholipid metabolism | 7 |
|  | Phenylalanine, tyrosine and tryptophan biosynthesis | 7 |
|  | Tricarboxylic acid cycle and glyoxylate/dicarboxylate metabolism | 6 |
|  | Arachidonic acid metabolism | 6 |
|  | Other amino acid | 6 |
|  | Acyl-CoA hydrolysis | 6 |
|  | Beta oxidation of even-chain fatty acids (mitochondrial) | 6 |
|  | Arginine and proline metabolism | 5 |
|  | Steroid metabolism | 5 |
|  | Nucleotide metabolism | 5 |
|  | Sphingolipid metabolism | 5 |
|  | Histidine metabolism | 5 |
|  | Alanine, aspartate and glutamate metabolism | 4 |
|  | Beta oxidation of odd-chain fatty acids (mitochondrial) | 4 |
|  | Beta oxidation of unsaturated fatty acids (n-9) (mitochondrial) | 4 |
|  | Amino sugar and nucleotide sugar metabolism | 4 |
|  | Omega-3 fatty acid metabolism | 4 |
|  | Fatty acid elongation (odd-chain) | 3 |
|  | O-glycan metabolism | 3 |
|  | Pool reactions | 3 |
|  | Glycerolipid metabolism | 3 |
|  | Formation and hydrolysis of cholesterol esters | 3 |
|  | Transport, Golgi apparatus | 3 |
|  | Fatty acid transfer reactions | 2 |
|  | Transport, lysosome to ER | 2 |
|  | Tryptophan metabolism | 2 |
|  | Glycosphingolipid biosynthesis-ganglio series | 2 |
|  | Linoleate metabolism | 2 |
|  | Glucocorticoid biosynthesis | 2 |
|  | Cysteine and methionine metabolism | 2 |
|  | Androgen metabolism | 2 |
|  | Folate metabolism | 2 |
|  | Pyruvate metabolism | 1 |
|  | Glutathione metabolism | 1 |
|  | Transport, nuclear | 1 |
|  | Protein modification | 1 |
|  | Oxidative phosphorylation | 1 |
|  | Pyrimidine metabolism | 1 |
|  | Transport, Golgi to lysosome | 1 |
|  | Carnitine shuttle (mitochondrial) | 1 |
|  | Galactose metabolism | 1 |
|  | Cholesterol biosynthesis 2 | 1 |
|  | Vitamin D metabolism | 1 |
|  | Beta oxidation of even-chain fatty acids (peroxisomal) | 1 |
|  | Pentose phosphate pathway | 1 |
|  | ROS detoxification | 1 |
|  | Omega-6 fatty acid metabolism | 1 |
